# Malignant Disease of the Larynx

**Published:** 1905-03

**Authors:** 


**Malignant Disease of the Larynx.** By PHILIP R. W. DE SANTI. F.R.C.S. Pp. i., 107. London: Baillière, Tindall & Cox. 1904.

One of the main objects of this book is to place before the profession the views held in England as to the correct operative treatment of laryngeal cancer—views which have not so far been sufficiently understood or appreciated abroad. There is no room for doubting that the author has been successful in carrying out this object, and we have in a very readable form one of the most succinct yet complete and reliable summaries of the position of British surgery in regard to laryngeal cancer. The earlier sections, dealing with etiology and pathological anatomy, are good, the former because it is so freely confessed that the etiology is unknown, and various unsupported hypotheses are not put forward as probable causes; the latter because it has been the subject of careful investigation by the author himself. The sections dealing with diagnosis and the indications for various operative measures are accurate, and fully represent the best thoughts of the most expert operators in this department of surgery.

